# Music Is Healing: Developing a Music Module for a Pain Management Intervention for African American Older Adults

**DOI:** 10.1093/geroni/igaa057.2967

**Published:** 2020-12-16

**Authors:** Mary Janevic, Sheria Robinson-Lane, Afton Hassett, Rebecca Courser

**Affiliations:** 1 University of Michigan School of Public Health, Ann Arbor, Michigan, United States; 2 University of Michigan, Ann Arbor, Michigan, United States

## Abstract

Music has a known analgesic effect. Our multidisciplinary team is developing a music-focused module for Positive STEPS, a pain self-management intervention based on principles of positive psychology. The priority population is African American older adults with disabling chronic pain. Positive STEPS is delivered via website and phone calls from community health workers. To inform program design, we conducted two focus groups with older adults in Detroit (n=16; 100% female and African American; 75% age 70+). All participants said they would enjoy using music to cope with pain. Content analysis revealed the following themes regarding music for pain management: it elicits positive memories, reduces stress, motivates exercise and daily activities, and promotes relaxation. Participants offered ideas for music-focused activities, including learning about unfamiliar genres and using music for meditation/relaxation. Findings will inform the design of a new music module, to be pilot-tested for its effect on participant engagement and pain-related outcomes.

